# Case Series: Two Cases of an Atypical Presentation of Oral Granular Cell Tumour

**DOI:** 10.1155/2012/159803

**Published:** 2012-12-26

**Authors:** Emma Cole, Naomi Rahman, Roger Webb

**Affiliations:** Department of Oral and Maxillofacial Surgery, Queen Mary's Hospital, Frognal Avenue, Sidcup, Kent DA14 6LT, UK

## Abstract

This paper describes two cases of oral granular cell tumours with an atypical clinical presentation; both are in females aged between 45 and 63 years of age. Granular cell tumours are unusual soft tissue neoplasm of neural or Schwann cell origin. Oral GCTs usually present clinically as pink or yellow small sessile lesions. GCTs are usually benign in nature; however they can present in a malignant form in fewer than 2% of cases. In benign cases treatment is surgical and usually curative with extremely low recurrence rates.

## 1. Introduction

A granular cell tumour (GCT) is an uncommon soft tissue neoplasm, first discovered in 1926 by Abrikossoff who termed it a “Myloblastenmyome” as it was postulated to be of myogenic origin [1]. It is now thought to be of neural or Schwann cell origin this is supported by the positive staining of GCTs for S-100 as outside the central nervous system S-100 is found only in Schwann cells [2]. They are estimated to account for 0.5% of all soft tissue tumours [3] and are two to three times more prevalent in females [3]. GCTs can occur at any age, but are most common in those in their fourth to sixth decades of life [4] and are uncommon in children [5].

These tumours can occur in a wide variety of anatomical sites including the orbit, larynx, parotid gland, breast, gastrointestinal tract, reproductive systems, and peripheral and cranial nerves. GCTs-present most frequently in the head and neck region, with over 50% of lesions occurring in this region [6]; the commonest site of presentation in this region is the tongue.

GCTs in the oral cavity usually present clinically as pink or yellow sessile lesions which are small, solitary, painless, and covered by intact mucosa [7]. GCT are usually benign in nature; however they can present in a malignant form in fewer than 2% of cases. In benign cases treatment is surgical and usually curative with extremely low recurrence rates [4].

This paper describes unusual presentation of two cases of oral GCT.

**Case 1**

**Figure 1:**
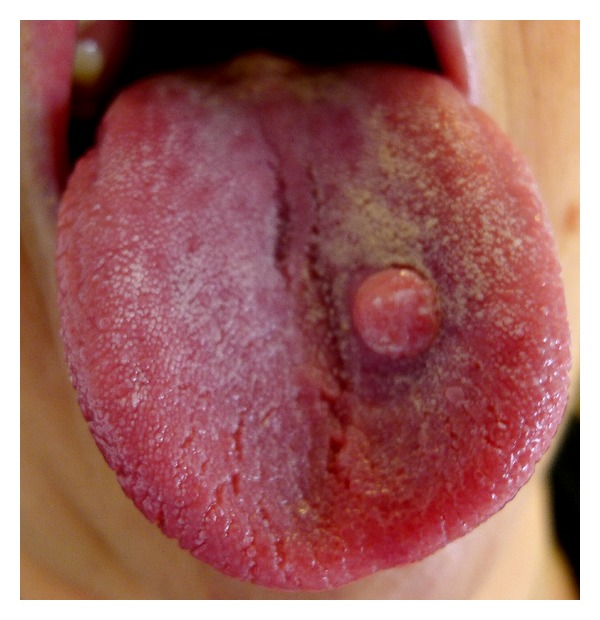
Intraoral photo of the granular cell tumour affecting the dorsum of the tongue.

A 63-year-old white Caucasian female with a three-month history of a painful, rapidly growing swelling on her tongue was referred to Department of Oral Maxillofacial Surgery in Kent, UK. She had a 40-year smoking history and was taking nicorandil. There was no evidence of extraoral pathology; intraorally the lesion presented as a firm, pedunculated, polypoid lesion with a flat surface on the dorsal surface of the tongue to the left of the midline, measuring 5 mm in diameter. The lump was tender to palpation and had an unusual hyperkeratotic surface (Figure 1).

**Figure 2:**
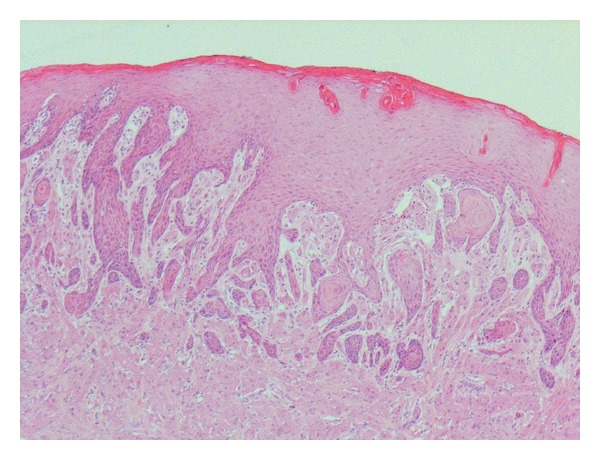
Low power view of oral granular cell tumour stained with hematoxylin and eosin.

**Figure 3:**
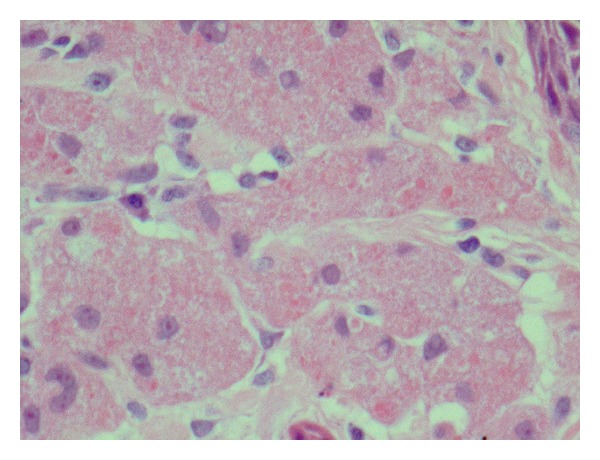
High power view of oral granular cell tumour stained with hematoxylin and eosin.

An excisional biopsy was performed under local anaesthesia and the specimen submitted for histological analysis (Figures 2 and 3). The histopathology report revealed a polypoid area in the centre of the biopsy composed of closely packed rounded cells with eosinophilic granular cytoplasm. The surface epithelium showed pseudoepitheliomatous hyperplasia. On immunostaining the epithelial cells were positive for CK5/6 and the stromal cells positive for S-100. The final diagnosis was a benign granular cell tumour. The patient now attends six monthly reviews and has had no recurrence.

**Case 2**

**Figure 4:**
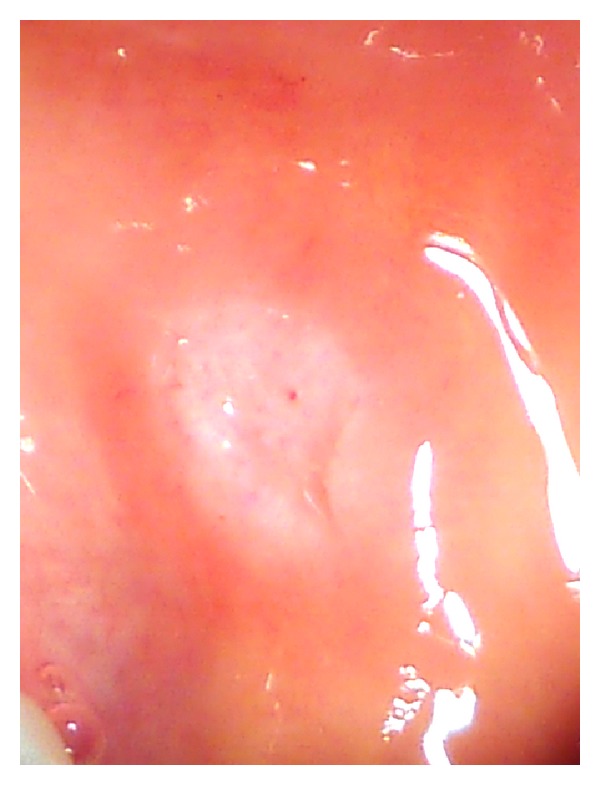
Intraoral photo of the granular cell tumour affecting the buccal mucosa.

A 45-year-old Caucasian female was referred to the department regarding an asymptomatic, raised white patch in her left buccal mucosa. Medically the patient was fit and well, however was a smoker. Extraorally no pathology was detected intraorally the lesion presented as a thick, raised, white plaque, fixed to underlying tissues and measuring 6 mm in diameter (Figure 4).

An incisional biopsy was performed under local anaesthetic and the specimen sent for histological analysis. This showed markedly hyperkeratotic, acanthotic squamous epithelium with intraepithelial cells containing granular cytoplasm.

**Figure 5:**
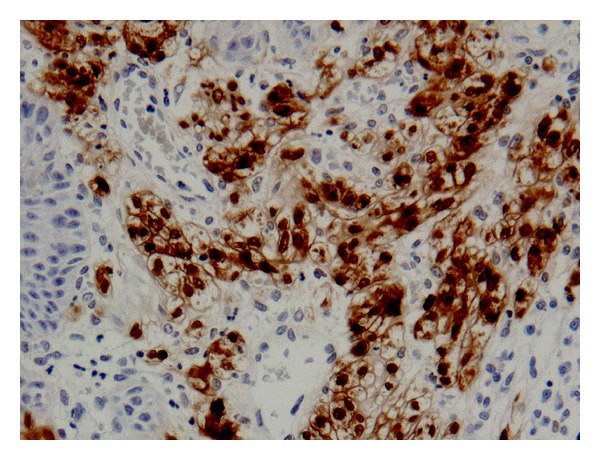
Immunoreactivity of the lesion for S-100 protein.

Immunocytochemistry showed a strong positive reaction for S-100. The initial report was suggestive of a granular cell tumour; however the biopsy was superficial, and a repeat excisional biopsy was performed. The results of this confirmed the diagnosis of a granular cell tumour; the immunohistochemistry showed that the cells were positive for S-100, but negative for Melan A, MNF-116, and CD68, consistent with a granular cell tumour (Figure 5).

## 2. Discussion

Granular cell tumor is an uncommon soft tissue neoplasm. Generally it is seen as a solitary asymptomatic nodule less than 3 cms in size involving the subcutaneous or submucosal tissues. The nodular mass is hard in consistency and generally reveals an intact overlying epithelium. This paper presents two cases of atypical presentation of such tumors. The first case showed an intraoral lesion that presented as a firm, pedunculated, polypoid lesion with a flat surface on the dorsal surface of the tongue. It was tender and had an unusual hyperkeratotic surface. Excisional biopsy was performed, and it confirmed the diagnosis. The second case showed an intraoral lesion that was thick, raised with a white plaque, and fixed to the underlying tissue. Incisional biopsy was performed and confirmed the diagnosis.

Clinical diagnosis of GCT is difficult due to the similarity in presentation with many other epithelial lesions; differential diagnosis may include fibromas, lipomas, neurofibromas, dermoid cyst, pleomorphic adenoma of minor salivary glands, and neuromas. In order to obtain a definitive diagnosis of these lesions, histological examination is necessary including monoclonal antibody S-100 staining. 

The optimal treatment is by local conservative surgical excision with a margin of soft tissue; when this is performed satisfactorily, recurrence rates are low. This treatment has been carried out in both of the previous cases, and recurrence has not occurred.
